# Mapping Network Motif Tunability and Robustness in the Design of Synthetic Signaling Circuits

**DOI:** 10.1371/journal.pone.0091743

**Published:** 2014-03-18

**Authors:** Sergio Iadevaia, Luay K. Nakhleh, Robert Azencott, Prahlad T. Ram

**Affiliations:** 1 Department of Systems Biology, The University of Texas MD Anderson Cancer Center, Houston, Texas, United States of America; 2 Department of Computer Science, Rice University, Houston, Texas, United States of America; 3 Department of Mathematics, University of Houston, Houston, Texas, United States of America; University of Georgia, United States of America

## Abstract

Cellular networks are highly dynamic in their function, yet evolutionarily conserved in their core network motifs or topologies. Understanding functional tunability and robustness of network motifs to small perturbations in function and structure is vital to our ability to synthesize controllable circuits. In establishing core sets of network motifs, we selected topologies that are overrepresented in mammalian networks, including the linear, feedback, feed-forward, and bifan circuits. Static and dynamic tunability of network motifs were defined as the motif ability to respectively attain steady-state or transient outputs in response to pre-defined input stimuli. Detailed computational analysis suggested that static tunability is insensitive to the circuit topology, since all of the motifs displayed similar ability to attain predefined steady-state outputs in response to constant inputs. Dynamic tunability, in contrast, was tightly dependent on circuit topology, with some motifs performing superiorly in achieving observed time-course outputs. Finally, we mapped dynamic tunability onto motif topologies to determine robustness of motif structures to changes in topology and identify design principles for the rational assembly of robust synthetic networks.

## Introduction

Intracellular networks are complex systems that coordinate the information flow from the extracellular environment into the cell to elicit appropriate gene regulatory and metabolic responses. The complexity of signaling, metabolic and gene regulatory networks arises from the extraordinary variety of molecular mechanisms that have evolved to ensure that these systems have the necessary robustness to adapt to environmental changes and compensate for intracellular perturbations [1]–[2]. Bypasses, redundancies, and regulatory loops are integrated at multiple levels to form highly interconnected webs of protein interactions that reliably regulate various cellular functions [3].

Dissecting the complexity of conserved cellular networks is perhaps one of the most challenging tasks in systems biology. Investigating the concept of network motifs as simple building blocks within larger networks [4] revealed that such motifs occur in biology, engineering, and ecology networks much more frequently than they occur in randomized networks. Further analysis of these conserved motif structures revealed associations with specific biological functions, such as robust dual-time switches [5], dynamics expression programs and responses to external signals [6], tunable oscillations [7], and biochemical adaptation [8]. We will interchange the words “motif” and “network motif” throughout the manuscript.

These studies posed the basis for the central hypothesis that only specific network motifs can underlie observed biological functions, whereas other circuit topologies do not have the ability to generate particular cellular outputs. However, even simple network motifs can exhibit wide ranges of static and dynamic behaviors, depending on the initial cellular state and the rate of information flow across the circuit [9]–[14]. Therefore, understanding whether certain network topologies preferentially underlie functionally associated cellular responses remains an open question.

Robustness of conserved signaling networks is attained through complex webs of protein interactions, which promote stability and redundancy, and through modularity, which may insulate functional properties and prevent failure from spreading across the network [15]. Robustness of core signaling motifs, however, cannot depend on modularity but rather must be an emergent property of the particular network topology or structure. Hence, understanding how robustness of network motifs in generating desired output responses correlates to the topology of particular motifs of interest represents another central yet open question to be addressed in the analysis of simple signaling circuits.

In this work, we used a computational approach that integrated ordinary differential equations (ODEs) with particle swarm optimization (PSO) to quantify tunability and robustness of network motifs to attain relevant input-output signaling responses. The ODE approach is commonly employed to describe the steady-state and transient behavior of network motifs in response to constant inputs [16]. PSO was used to identify sets of model parameters representative of the different dynamic behaviors exhibited by the various network motifs. We selected PSO because of its superior ability to converge to more optimal solutions compared with other optimization algorithms [17], [18]. In establishing core sets of network motifs, we selected simple topologies that are overrepresented in mammalian signaling networks, such as the linear, feedback, feed-forward, and bifan circuits [16], [19]. Our computational results suggested that static tunability of the network motifs is insensitive to the circuit topology, as all the signaling motifs displayed similar ability to attain predefined static outputs in response to constant inputs. Dynamic tunability, in contrast, was tightly dependent on the circuit topology, with some motifs performing superiorly in achieving observed time-course outputs. We further mapped functional tunability onto topologies of signaling motifs and determined the robustness of network structures to changes in topology to identify design principles for the rational assembly of robust synthetic networks.

## Results

### Core sets of motif topologies

**Figure 1:**
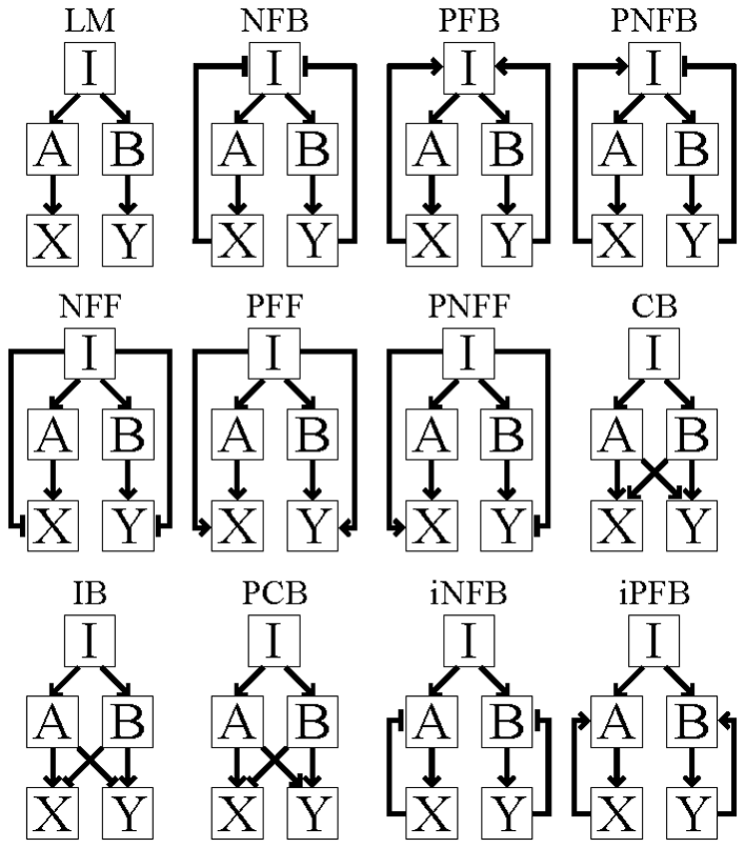
Motifs topologies overrepresented in mammalian signaling networks. In all of the motifs, “I” is the input source that activates signals across the networks; “A, B” are intermediate components; and “X, Y” are downstream effectors. LM: linear motif; NFB: negative feedback; PFB: positive feedback; PNFB: positive-negative feedback; NFF: negative feed-forward; PFF: positive feed-forward; PNFF: positive-negative feed-forward; CB: coherent bifan; IB: incoherent bifan; PCB: partially coherent bifan; iNFB: isolated negative feedback; iPFB: isolated positive feedback.

The motif topologies [16], [19] overrepresented in mammalian signaling networks that we examined are shown in Figure 1. In all of the motifs, “I” is the input source that activates signals across the networks; “A, B” are intermediate components; and “X, Y” are downstream effectors. In the *linear motif* (LM), signals are transduced from the input source I to the downstream effectors X, Y via linear cascades through A, B, respectively. In the *negative feedback* (NFB), *positive feedback* (PFB), *positive-negative feedback* (PNFB), *isolated negative feedback* (iNFB), and *isolated positive feedback* (iPFB) *motifs*, information from the downstream effectors can be transmitted back to either the input source I (feedback) or to the intermediate molecules A, B (isolated feedback). In the *negative feed-forward* (NFF), *positive feed-forward* (PFF), and *positive- negative feed-forward* (PNFF) *motifs*, signals from the input source directly modulate the downstream effectors X, Y and the intermediates A, B. Finally, in the *coherent bifan* (CB), *incoherent bifan* (IB), and *partially coherent bifan* (PCB) *motifs*, the downstream effectors X, Y are regulated by both intermediates A, B. All of the network motifs have in common the same core structure, which is that of the linear motif. The interactions that augmented the linear motif core structure can be any combination of positive or negative effects. Each motif topology can be obtained from any one of the other structures, where the occurrence of “mutations” can add or remove interactions within motif components (Table S1 in file S1).

### Functional tunability of network motifs

Functional tunability was defined as the ability to tune the model parameters of each network motif to attain predefined static and transient outputs in response to constant input stimulations. To quantify and rank motif tunability of the various motifs, we implemented ODE modeling (Table S2 in file S1) to predict the output responses of activated X and Y (X* and Y*, respectively) and to determine whether specific topologies performed superiorly in achieving predefined output objectives. Motifs tuned to achieve an objective were termed “plastic” if easily tuned or termed “rigid” if tuned with difficulty.

**Figure 2:**
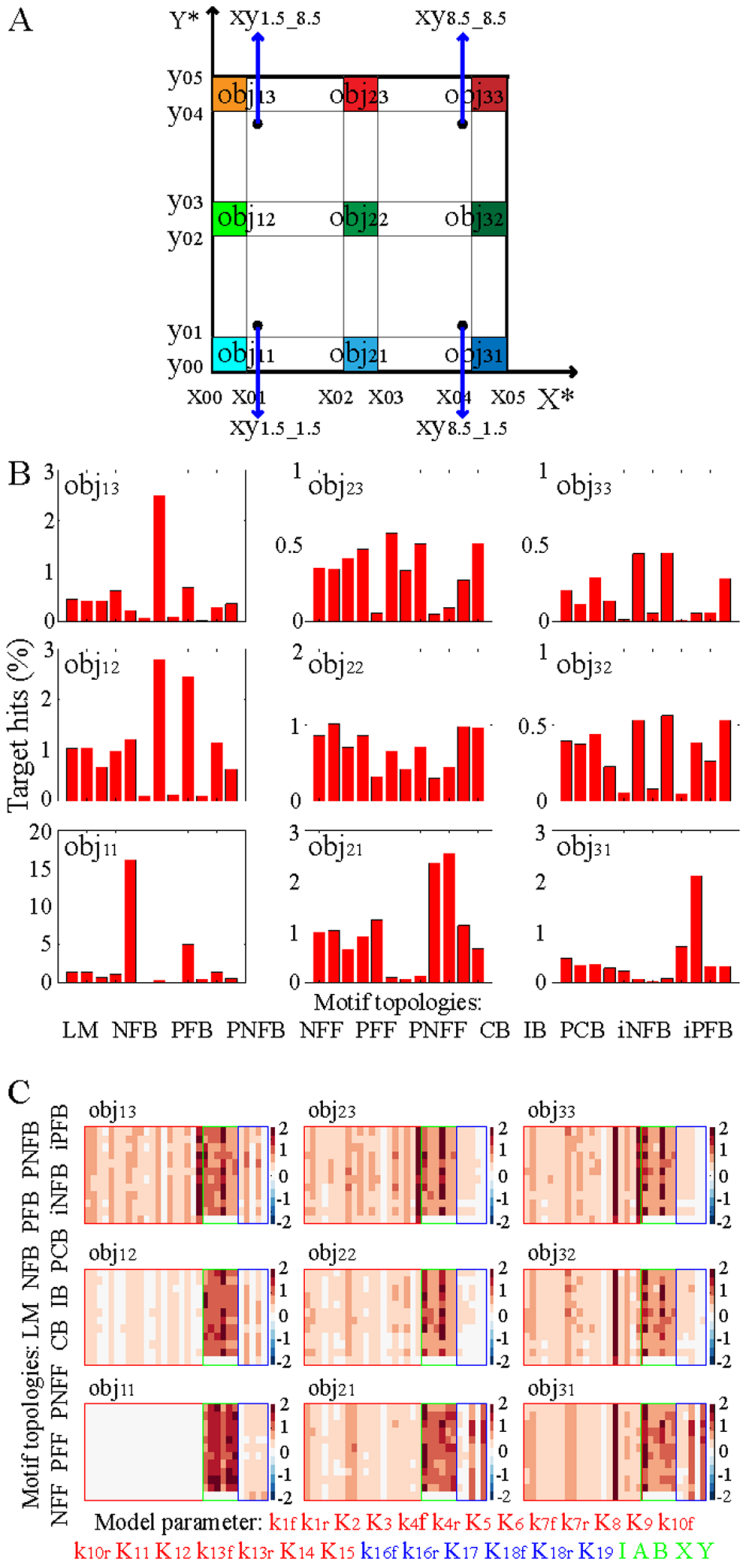
Functional tunability of network motifs to attain static output objectives. A: Predefined steady-state objective: [x_00_ x_01_] = [0 1], [x_02_ x_03_] = [4.5 5.5], [x_04_ x_05_] = [9 10]; [y_00_ y_01_] = [0 1], [y_02_ y_03_] = [4.5 5.5], [y_04_ y_05_] = [9 10]; obj_11_, obj_12_, and obj_13_: X^*^ is low and Y^*^ is low, intermediate, or high; obj_21_, obj_22_, and obj_23_: X^*^ is intermediate and Y^*^ is low, intermediate, or high; obj_31_, obj_32_, and obj_33_: X^*^ is high and Y^*^ is low, intermediate, or high. PSO implementation: steady-state levels of motif output responses were arbitrarily set to the following values: [X^*^ Y^*^] = [1.5 1.5] (xy_1.5_1.5_); [X^*^ Y^*^] = [1.5 8.5] (xy_1.5_8.5_); [X^*^ Y^*^] = [8.5 1.5] (xy_8.5_1.5_); and [X^*^ Y^*^] = [8.5 8.5] (xy_8.5_8.5_). Model parameters of all motifs were initialized to identical values as follows: 1) PSO was pre-implemented to identify model parameters that generated such static outputs for the linear motif; 2) these parameters were used for the core structure of all other network motifs; 3) the kinetic constants of the additional reactions were set to zero. B: Motif tunability to static output objectives obtained through random sampling of model parameters. C: Motif tunability to static output objectives obtained through PSO sampling of model parameters necessary for ODE implementation. Particle positions were initialized to the point xy_1.5_1.5_. CV was defined as the ratio between the standard deviation and the mean computed across the 100 parameter sets identified by using PSO. Given the 12 motif topologies, 9 objective areas, and 100 sets of identified parameter, PSO was implemented 10,800 times.

We compared the motif performance in attaining static outputs by quantifying the motif tunability in generating steady-state responses that reached low, intermediate, or high levels of X* and Y*. Figure 2A shows the static output objectives to be achieved by the output responses X^*^ and Y^*^ for the various network motifs. Objective areas obj_11_, obj_22_, and obj_33_ represent states in which both output responses attained comparable levels that were low, intermediate, or high. The other objective areas define states in which X^*^ and Y^*^ reached different steady-state levels, as indicated on the graph. We quantified and ranked functional tunability of signaling motifs to reach particular steady-state outputs by using two different approaches, described as follows.

In the first approach, we randomly sampled the model parameters from predefined ranges of values (Methods section). We then implemented ODE modeling by using the same set of randomly sampled parameters across all of the network motifs and determined the static output responses attained by the various network motifs. We repeated these steps independently 100,000 times and ranked tunability by computing the percentage of target hits (TH). TH was defined as the percentage of times that the steady-state values of X^*^ and Y^*^ were located in one of the predefined objective areas over the total number of attempts. The computational results, shown in Figure 2B, indicated that TH decreased as the motif output responses reached higher steady-state levels. Hence, full motif activation—in which X^*^ and Y^*^ attained high levels—required initial cellular states and rates of information flow such that strong signals were rapidly transduced across the networks. If the initial concentrations of signaling molecules or the kinetic rate constants were not appropriately sampled, weaker signals were transduced slower, and the steady-state values of X^*^ and Y^*^ remained low. Therefore, for any given structure, full motif activation was found to be less likely to occur. The results also suggested that full activation of network motifs was facilitated in CB, PFF, PFB, and LM topologies, whereas low network activation was more likely to occur in NFF, IB, and NFB motifs.

In the second approach, we integrated ODE modeling with PSO to identify initial cellular states and rates of information flow that generated steady-state output responses that were located in each of the nine objective areas (Methods section). Particle positions of each motif—i.e., the model parameters—were initialized to identical values such that the levels of X^*^ and Y^*^ attained static values that were outside the objective areas. These levels were denoted as xy_1.5_1.5_, xy_1.5_8.5_, xy_8.5_8.5_, and xy_8.5_8.5_, respectively (Fig. 2A). Particle velocities—i.e., the extent to which the model parameters were changed—were randomly sampled. PSO was implemented to minimize the static square error and to identify 100 sets of model parameters that generated steady-state output responses that were located in each objective area. Motif tunability was ranked by using the coefficients of variation (CVs) of the identified model parameters. Bigger CVs correlated with higher flexibility with which parameters could be tuned to attain wider ranges of steady-state outputs and, in turn, superior motif plasticity. Figure 2C and Figure S1 (in file S1) show the heatmaps of the CVs of the motif parameters that generated static levels of X^*^ and Y^*^ that attained the predefined objective areas. The computational results suggested that the variability of the parameters defining the core structure common to all motifs was very small when the values of static output responses to be attained were closer to those used to initialize the model parameters for all motifs. However, the parameter variability increased as the steady-state objective values were further away from the initial levels of X^*^ and Y^*^. Therefore, the contribution of the core structure to the overall motif tunability increased with the distance between initial and static output objectives for all motifs.

The results also indicated that the variability of the parameters that define the common core structure is smaller than the variability of the parameters defining the reactions that augment the core structure, regardless of the choice of the particular objective areas to be achieved and the initial levels of X^*^ and Y^*^. Therefore, the existence of additional protein interactions confers to feedback, feed-forward, and bifan topologies a small gain in plasticity to attain steady-state output objectives compared with the linear motif.

Finally, the results suggested that higher tunability of X^*^ and Y^*^ deactivation rates—k_12_ and k_15_—could facilitate full motif activation independently on the strength and speed of signal transduction across the various motifs. Therefore, full network activation seemed to be preferentially accomplished through inhibition of X^*^ and Y^*^deactivation rather than via modulation of the overall signal flow across the networks.

The computational results obtained by using these two different approaches converged toward a unified interpretation: all the signaling motifs displayed comparable functional tunability in reaching predefined static output objectives in response to constant inputs. Therefore, the static tunability of the network motifs appeared to be insensitive to the particular topology of the circuit of interest.

**Figure 3:**
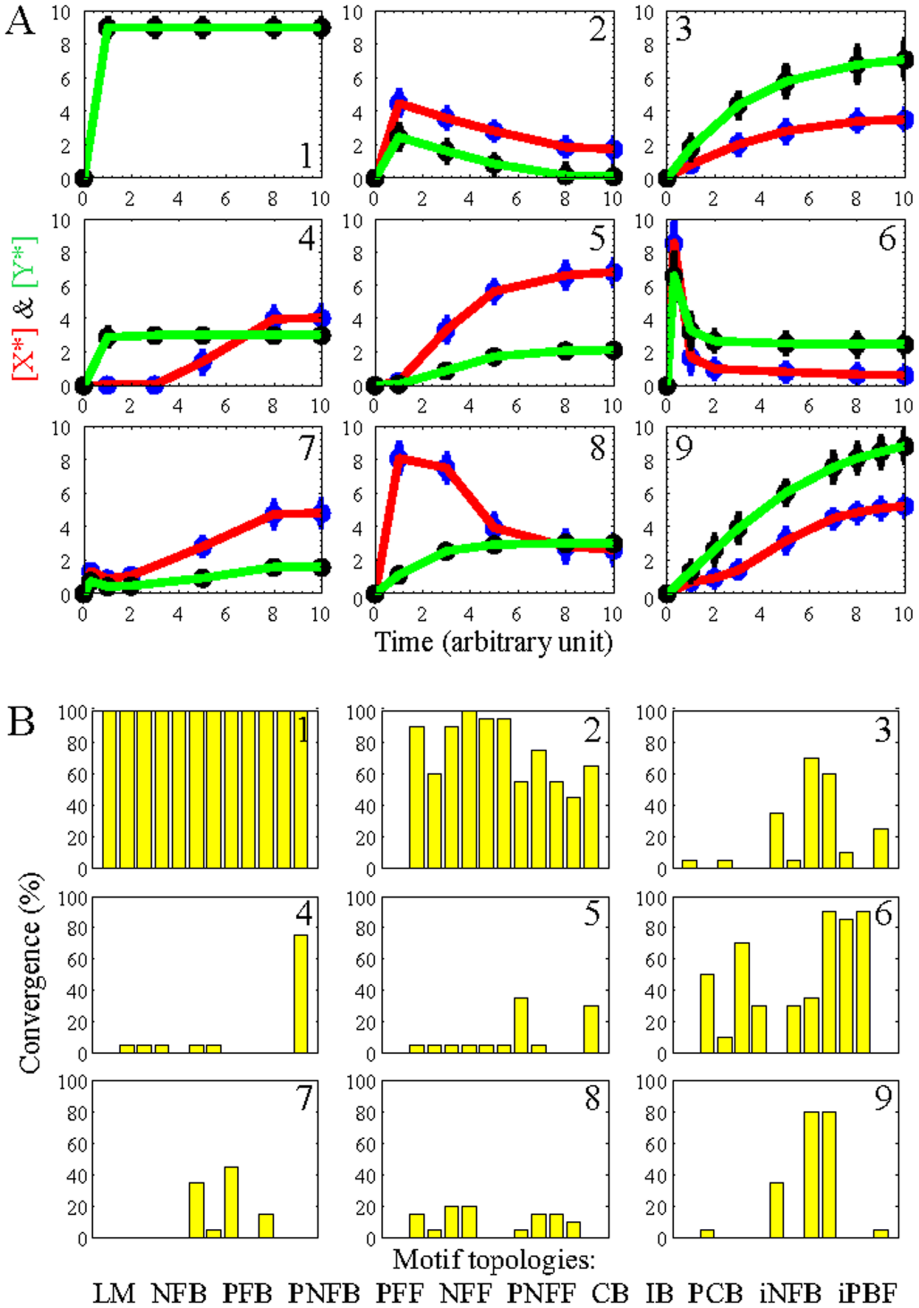
Motif tunability to transient output objectives. A: Predefined time-courses and B: convergence percentage of network motifs to transient output objectives for: 1) fast; 2) slowly decaying; 3) asymptotic; 4) rapid and delayed; 5) sigmoidal; 6) pulse; 7) biphasic; 8) rapid increasing-slow decaying and asymptotic; and 9) multi-static responses. Given the 12 motif topologies, 9 time-course objectives, and 20 attempts to identify the model parameter, PSO was implemented 2,160 times.

To quantify functional tunability of signaling motifs to transient output objectives, we compared the motif performance in generating output responses of X^*^ and Y^*^ that resembled those of commonly observed time-courses from biological signaling networks [20]–[23], as shown in Figure 3A. We implemented the integrative PSO-ODE approach to compute the transient output responses attained by the various networks when the particle positions of all motifs were initialized to identical values. In this regard, we set the values of the kinetic constants of the reactions that augment the core structure common to all motifs to zero to reduce the topology of all motifs to that of the core structure. We then randomly sampled the particle positions of the common core structure from the uniform distribution and initialized the values of particle velocities to zero. To rank motif tunability, we initialized the model parameters by using 20 randomly sampled sets and implemented PSO 20 times to identify for each motif 20 sets of model parameters that generated the predefined transient output responses. Since not all of the motifs displayed the ability to produce such objective responses for each PSO run, functional tunability was ranked by computing the convergence percentage with which each motif could generate output responses that matched those of the transient output objectives.

Figure 3B shows the functional tunability of network motifs in generating signaling response that attained predefined output objectives. The computational results indicated that: 1) all the motifs had the same plasticity in generating fast responses; 2) NFF, PFF, PNFF, NFB, and PNFB were more plastic motifs in producing slowly decaying responses; 3) CB and IB were more plastic structures in displaying asymptotic responses; 4) iPFB displayed superior plasticity in generating rapid and delayed responses; 5) CB and iPFB were more plastic motifs in producing sigmoidal responses; 6) IB, iNFB, and PCB were more plastic structures in generating pulse responses; 7) CB and NFF displayed superior plasticity in generating biphasic responses; 8) PNFB and PFF exhibited superior plasticity in producing rapid increasing-slow decaying and asymptotic responses; and 9) CB and IB were more plastic motifs in displaying multi-static responses.

Overall, CB was the most plastic motif across the whole set of predefined output responses (8 of 9 times), whereas LM was the most rigid motif (2 of 9 times). Moreover, bifan motifs displayed on average superior plasticity across the whole set of signaling output responses (7 of 9 times) compared with the feedback motifs (6.67 of 9 times), feed-forward motifs (6.33 of 9 times), and isolated feedback motifs (5 of 9 times). As the signaling motifs displayed different functional abilities to attain predefined transient output objectives in response to constant inputs, the transient tunability of the network motifs appeared to be tightly dependent on the particular topology of the circuit of interest.

### Motif robustness to functional tunability

Since each motif topology can be obtained from that of other structures through occurrence of mutations that enable or impair additional interactions within motif components, motif robustness to functional tunability was defined as the motif ability to produce virtually identical signaling outputs when the interaction network that underlies the motif topology of interest is altered due to mutations. In this regard, we constructed a web of 81 motif topologies (Table S3 in file S1) that interconnected the linear, feedback, feed-forward, bifan, and isolated feedback motifs (Fig. S2 in file S1). This network was obtained by starting with the linear motif and allowing at most the occurrence of two mutations. Since all of the motif topologies have similar functional tunability in producing static output responses, we focused our attention on the transient tunability and integrated ODE modeling with PSO to compute the convergence frequency of the 81 motifs in generate signaling time-courses that resembled those of the slowly decaying and pulse output objectives (Fig. 3A). To compute the convergence frequency, we implemented PSO 20 times and counted the number of times that a given motif could generate predefined transient output responses.

**Figure 4:**
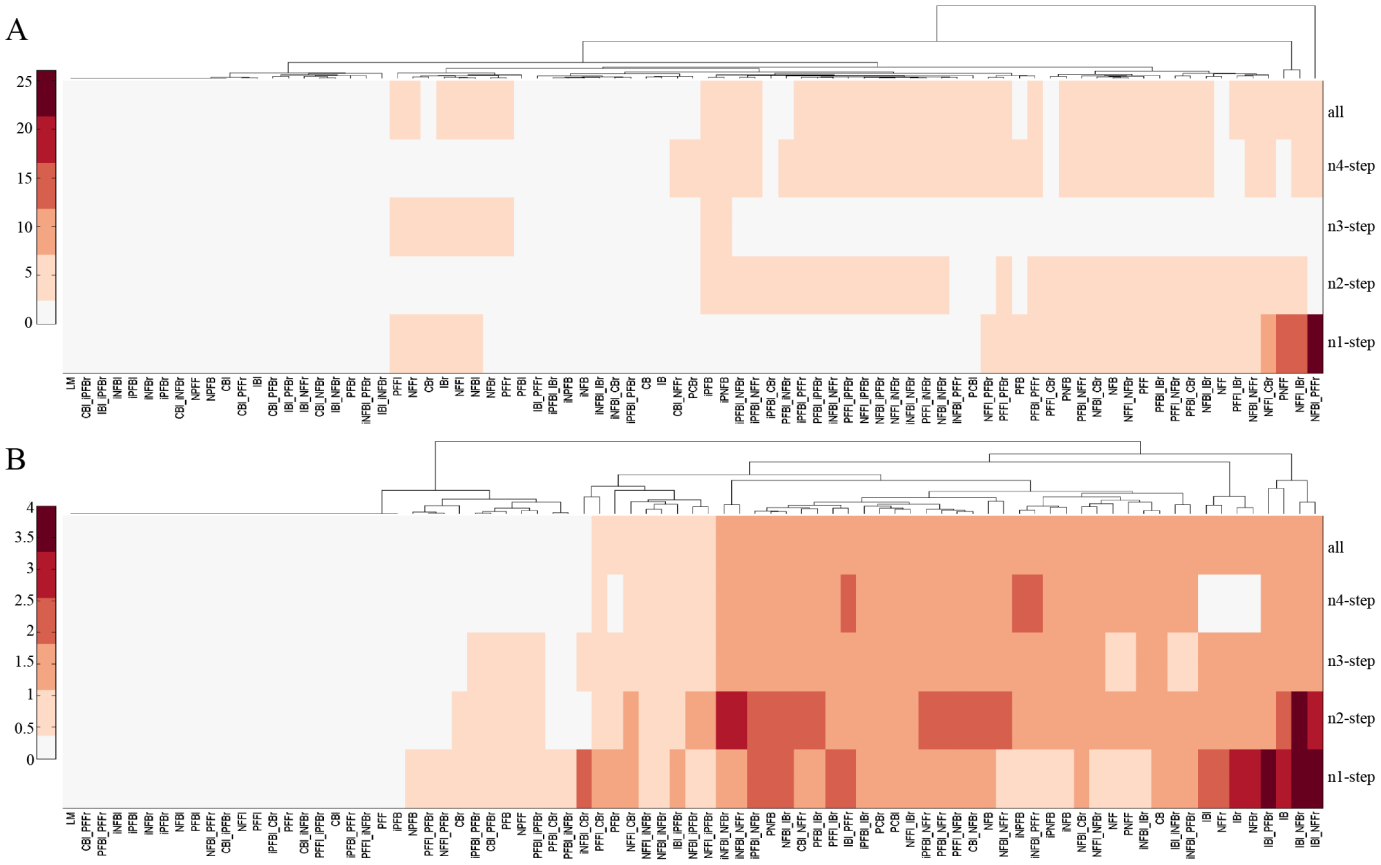
Robustness index heatmaps of the 81 motif topologies with respect to i-step neighbors. A: Robustness of functional tunability to slowly decaying responses. B: Robustness of functional tunability to pulse responses. Given the 81 motif topologies, 2 time-course objectives, and 20 attempts to identify the model parameter, PSO was implemented 3,240 times.

Standard robustness analysis of network topologies is generally focused on quantifying robustness of circuit structures with respect to their immediate neighbors. We extended this analysis beyond immediate neighbors and computed the robustness R(h,i) of motif topologies with respect to the 1-step, 2-step, 3-step, and 4-step neighbors and with respect to all other topologies that make up the web of networks (Methods section). Figure 4 shows the heatmaps of the robustness index R(h,i) of the 81 motif topologies with respect to their neighbors in producing cellular output responses that matched those of the slowly decaying and pulse output objectives. The computational results suggested that NFBl_PFFr (left negative feedback and right positive feed-forward), NFFl_IBr (left negative feed-forward and right incoherent bifan), and PNFF are optimal topologies to robustly and frequently generate slowly decaying signaling outputs with respect to their immediate neighbors (Fig. 4A). Moreover, while NFFl_IBr and PNFF displayed superior robustness compared with NFBl_PFFr with respect to their 2-step neighbors, the 3 topologies have similar robustness with respect to their 3-step neighbors and all of the other motif topologies, whereas NFBl_PFFr and NFFl_IBr exhibited superior robustness compared with PNFF with respect to their 4-step neighbors. Overall, NFFl_IBr and PNFF are more optimal topologies than NFBl_PFFr is because of their superior robustness with respect to 1-step and 2-step neighbors. The computational results also indicated that IBl_NFFr (left incoherent bifan and right negative feed-forward), IBl_NFBr (left incoherent bifan and right negative feedback), IB, and IBl_PFBr (left incoherent bifan and right positive feedback) are optimal topologies to robustly and frequently generate pulse signaling output responses with respect to their immediate neighbors (Fig. 4B). Furthermore, IBl_NFBr exhibited superior robustness compared with the other 3 topologies with respect to their 2-step neighbors, whereas the 4 topologies displayed similar robustness with respect to all of the other motifs as well as to their 3-step and 4-step neighbors. Overall, IBl_NFBr is more optimal than the other motifs are because of its superior robustness with respect to the immediate and 2-step neighbors.

## Discussion

Quantifying the functional tunability and robustness with which various motif topologies are able to generate biologically relevant output responses is instrumental to understanding overrepresentation of simple motifs in mammalian signaling networks. Although particular network topologies may be optimal to reliably generate a limited number of desired signaling outputs, other motifs may non-optimally produce wider ranges of biologically relevant cellular outcomes. There has been much work done on the design of synthetic circuits using different structural motifs to achieve specific objective functions to address important problem [24]–[26]. In this context, our integrative approach provides a useful tool with which to characterize regulatory properties of signaling motifs and to map superior mutational evolution of motifs onto their topology. Our integrative procedure may also provide a compendium of design principles for the rational assembling and engineering of synthetic networks that may robustly exhibit desired functional tunability when transfected into bacterial and yeast species.

## Methods

### Mass-action modeling

The dynamics of network motifs were described with use of mass-action models of ordinary differential equations (ODEs). The interaction networks of simple motifs were reconstructed as chemical reactions (Table S1 in file S1), which described the simplified mechanisms of activation and inhibition of signaling proteins. The chemical reactions were transformed into systems of coupled ODEs by assuming that the accumulation rate of the concentration of the *i* ^th^ signaling component was expressed as the difference between its net rates of production and consumption (Table S2 in file S1). ODE modeling was implemented using the implicit ode15s routine for stiff systems (Matlab R2008b, The MathWorks, Natick, MA) to predict the output responses of the various motifs and to determine whether motifs of interest could attain predefined static and transient output objectives. ODE implementation requires the selection of model parameters, which are the kinetic rate constants (*K's*) and the initial concentrations of inactive, active, and complex species (*C's*). Since the values of the rate constants and initial concentrations are largely unknown for virtually all biological systems, we arbitrarily selected ranges of values for the model parameters: K's 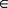 [K_min_ K_max_]  =  [0 100]; C's 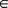 [C_min_ C_max_]  =  [0 10]. K_min_, K_max_, C_min_, and C_max_ are the minimum and maximum values of the kinetic rate constants and the initial protein concentrations, respectively. ODE modeling was implemented by either randomly sampling the parameter values within these ranges or using particle swarm optimization (PSO) because of its superior ability to converge to more optimal solutions compared with other optimization algorithms^17^.

### Random sampling

Model parameters were randomly sampled from the uniform distribution and converted into their final values as follows:
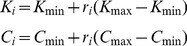
(1)


In equation 1, *r_i_* represents random numbers uniformly distributed in the interval [0 1].

### PSO

For details about PSO, see the methods described in previous publications [17], [18]. In our settings, the particle positions are the parameter values used in the ODE model to computationally generate the motif output responses X^*^ and Y^*^, and the particle velocities denote the extent to which the parameter values were iteratively changed. The steady-state fitness was defined as the distance between the centers of predefined objective areas (Figure 2A) and the ODE-predicted static values X^*^ and Y^*^, which was evaluated by using the static square error:
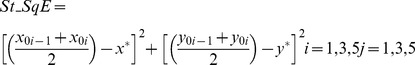
(2a)


In equation 2a, (x_0i-1_+x_0i_)/2 and (y_0i-1_+y_0i_)/2 represent the centers of the predefined objective areas. The transient fitness was defined as the distance between predefined and ODE-computed time-courses of X^*^ and Y^*^ (Figure 3A), which was evaluated by using the transient square error: 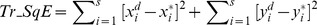
(2b)


In equation 2b, *x^d^_i_* and *y^d^_i_* are the predefined time-courses, respectively, whereas *s* represents the total number of data points that make up a time-course. Model parameters were initialized to identical values for all the network motifs and iteratively changed according to equation 2a until 1) the static values of X^*^ and Y^*^ were in the objective areas or 2) the time-courses of X^*^ and Y^*^ were within the error bars of the predefined transient objectives.

### Robustness

We quantified robustness of network motifs in the context of functional tunability by defining an index that provided a measure of how frequently and robustly various motif topologies could generate predefined output objectives. Only structures that could produce signaling responses with high robustness and convergence frequency were optimal. Non-optimal structures, on the contrary, generated signaling responses either with high robustness but low frequency or high frequency but low robustness. We defined the index *R*(*h,i*) as follows:
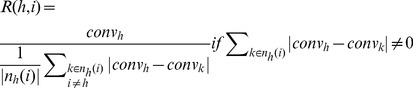
(3a)


In equation 3a, *i* represents different types of neighbors, which include 1-step or immediate neighbors (*i* = 1), 2-step (*i* = 2), 3-step (*i* = 3), and 4-step (*i* = 4) neighbors, *h* denotes a given motif topology, and *k* represents a neighbor topology of a particular motif. Moreover, *n_h_*(*i*) is the set of neighbors, and *conv_j_* (*j* = *h*,*k*) is the convergence frequency, which accounts for the number of times out of the 20 PSO runs that a motif topology could generate output responses that matched those of predefined objectives. Since *conv_h_*, |*conv_h_ – conv_k_*|, and |*n_h_*(*i*) | are positive integers, then the index *R*(*h,i*) increased when 1) the frequency of convergence of a given motif topology *h* was high; and 2) the difference between the convergence frequency of the structure of interest and those of the various i-step neighbors was small. When the sum of the difference between the convergence frequency of a given structure and those of the various i-step neighbors was zero, then the index *R*(*h,i*) was computed as follows:
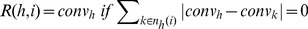
(3b)


Thus, if the convergence frequency of all motifs was low or zero, then the motif in question would barely be able to generate the predefined output objective or not be able to generate it at all and would be found to be not optimal. Conversely, if the convergence frequency of all motifs was high, then the motif would be found to be optimal.

## Supporting Information

File S1 Figure S1, Motifs tunability to static output objectives. Figure S2, Interaction network of 81 motif topologies that linked the linear, feedback, feed-forward, bifan, and isolated feedback motifs. Table S1, Reaction networks describing the dynamics of the signaling motif. Table S2, Ordinary differential equation (ODE) modeling describing the dynamics of the signaling motif. Table S3, Motif topologies that interconnected the linear, feedback, feed-forward, bifan, and isolated feedback motifs.
(PDF)
