# Supplementary material for: Mapping Network Motif Tunability and Robustness in the Design of Synthetic Signaling Circuits
Source: PLoS One. 2014 Mar 18;9(3):e91743. doi: 10.1371/journal.pone.0091743 (PMC3958390; doi:10.1371/journal.pone.0091743)
Supplement: File S1 — Figure S1, Motifs tunability to static output objectives. Figure S2, Interaction network of 81 motif topologies that linked the linear, feedback, feed-forward, bifan, and isolated feedback motifs. Table S1, Reaction networks describing the dynamics of the signaling motif. Table S2, Ordinary differential equation (ODE) modeling describing the dynamics of the signaling motif. Table S3, Motif topologies that interconnected the linear, feedback, feed-forward, bifan, and isolated feedback motifs. (PDF) [file pone.0091743.s001.pdf]

Mapping motif tunability and robustness in the design of synthetic signaling circuits

Sergio Iadevaia[1*], Luay K. Nakhleh[2], Robert Azencott[3] & Prahlad T. Ram[1*]

[1]Department of Systems Biology, The University of Texas MD Anderson Cancer Center, Houston, Texas, 77030, USA. [2]Department of Computer Science, Rice University, Houston, Texas, 77005, USA. [3]Department of Mathematics, University of Houston, Houston, Texas, 77004, USA.

* To whom correspondence should be addressed. Mailing address: 7435 Fannin St, Unit 950, P.O. Box 301429. E-mail: siadevai@mdanderson.org. or pram@mdanderson.org; phone: 1-713-563-4227; fax: 1-713-563-4235.

Contents

SM1. Supplementary Table I: Reaction networks describing the dynamics of the signaling motif.

SM2. Supplementary Table II: Ordinary differential equation (ODE) modeling describing the dynamics of the signaling motif.

SM3. Supplementary Table III: Motif topologies that interconnected the linear, feedback, feedforward, bifan, and isolated feedback motifs.

SM4. Supplementary Figure 1: Motifs tunability to static output objectives.

SM5. Supplementary Figure 2: Interaction network of 81 motif topologies that linked the linear, feedback, feed-forward, bifan, and isolated feedback motifs.

SM1. Supplementary Table I

Linear motif core structure	
$I + L \underset{k_1^r}{\overset{k_1^f}{\rightleftarrows}} IL \xrightarrow{K_2} I^* + L$	*LM Rx 1*
$I^* \xrightarrow{K_3} I$	*LM Rx 2*
$A + I^* \underset{k_4^r}{\overset{k_4^f}{\rightleftarrows}} AI \xrightarrow{K_5} A^* + I^*$	*LM Rx 3*
$A^* \xrightarrow{K_6} A$	*LM Rx 4*
$B + I^* \underset{k_7^r}{\overset{k_7^f}{\rightleftarrows}} BI \xrightarrow{K_8} B^* + I^*$	*LM Rx 5*
$B^* \xrightarrow{K_9} B$	*LM Rx 6*
$X + A^* \underset{k_{10}^r}{\overset{k_{10}^f}{\rightleftarrows}} XA \xrightarrow{K_{11}} X^* + A^*$	*LM Rx 7*
$X^* \xrightarrow{K_{12}} X$	*LM Rx 8*
$Y + B^* \underset{k_{13}^r}{\overset{k_{13}^f}{\rightleftarrows}} YB \xrightarrow{K_{14}} Y^* + B^*$	*LM Rx 9*
$Y^* \xrightarrow{K_{15}} Y$	*LM Rx 10*
Negative feedback additional reactions	
$I^* + X^* \underset{k_{16}^r}{\overset{k_{16}^f}{\rightleftarrows}} IX \xrightarrow{K_{17}} I + X^*$	*NFB Rx 11*
$I^* + Y^* \underset{k_{18}^r}{\overset{k_{18}^f}{\rightleftarrows}} IY \xrightarrow{K_{19}} I + Y^*$	*NFB Rx 12*
Positive feedback additional reactions	
$I + X^* \underset{k_{16}^r}{\overset{k_{16}^f}{\rightleftarrows}} IX \xrightarrow{K_{17}} I^* + X^*$	*PFB Rx 11*
$I + Y^* \underset{k_{18}^r}{\overset{k_{18}^f}{\rightleftarrows}} IY \xrightarrow{K_{19}} I^* + Y^*$	*PFB Rx 12*
Positive-negative feedback additional reactions	
$I + X^* \underset{k_{16}^r}{\overset{k_{16}^f}{\rightleftarrows}} IX \xrightarrow{K_{17}} I^* + X^*$	*PNFB Rx 11*
$I^* + Y^* \underset{k_{18}^r}{\overset{k_{18}^f}{\rightleftarrows}} IY \xrightarrow{K_{19}} I + Y^*$	*PNFB Rx 12*
Negative feedforward additional reactions	
$X^* + I^* \underset{k_{16}^r}{\overset{k_{16}^f}{\rightleftarrows}} XI \xrightarrow{K_{17}} X + I^*$	*NFF Rx 11*
$Y^* + I^* \underset{k_{18}^r}{\overset{k_{18}^f}{\rightleftarrows}} YI \xrightarrow{K_{19}} Y + I^*$	*NFF Rx 12*
Positive feedforward additional reactions	
$X + I^* \underset{k_{16}^r}{\overset{k_{16}^f}{\rightleftarrows}} XI \xrightarrow{K_{17}} X^* + I^*$	*PFF Rx 11*

$Y + I^* \underset{k_{18}^r}{\overset{k_{18}^f}{\rightleftarrows}} YI \xrightarrow{K_{19}} Y^* + I^*$	*PFF Rx 12*
Positive-negative feedforward additional reactions	
$X + I^* \underset{k_{16}^r}{\overset{k_{16}^f}{\rightleftarrows}} XI \xrightarrow{K_{17}} X^* + I^*$	*PNFF Rx 11*
$Y^* + I^* \underset{k_{18}^r}{\overset{k_{18}^f}{\rightleftarrows}} YI \xrightarrow{K_{19}} Y + I^*$	*PNFF Rx 12*
Coherent bifan additional reactions	
$Y + A^* \underset{k_{16}^r}{\overset{k_{16}^f}{\rightleftarrows}} YA \xrightarrow{K_{17}} Y^* + A^*$	*CB Rx 11*
$X + B^* \underset{k_{18}^r}{\overset{k_{18}^f}{\rightleftarrows}} XB \xrightarrow{K_{19}} X^* + B^*$	*CB Rx 12*
Incoherent bifan additional reactions	
$Y^* + A^* \underset{k_{16}^r}{\overset{k_{16}^f}{\rightleftarrows}} YA \xrightarrow{K_{17}} Y + A^*$	*CB Rx 11*
$X^* + B^* \underset{k_{18}^r}{\overset{k_{18}^f}{\rightleftarrows}} XB \xrightarrow{K_{19}} X + B^*$	*CB Rx 12*
Partially coherent bifan additional reactions	
$Y + A^* \underset{k_{16}^r}{\overset{k_{16}^f}{\rightleftarrows}} YA \xrightarrow{K_{17}} Y^* + A^*$	*PCB Rx 11*
$X^* + B^* \underset{k_{18}^r}{\overset{k_{18}^f}{\rightleftarrows}} XB \xrightarrow{K_{19}} X + B^*$	*PCB Rx 12*
Isolated negative feedback additional reactions	
$A^* + X^* \underset{k_{16}^r}{\overset{k_{16}^f}{\rightleftarrows}} AX \xrightarrow{K_{17}} A + X^*$	*iNFB Rx 11*
$B^* + Y^* \underset{k_{18}^r}{\overset{k_{18}^f}{\rightleftarrows}} BY \xrightarrow{K_{19}} B + Y^*$	*iNFB Rx 12*
Isolated positive feedback additional reactions	
$A + X^* \underset{k_{16}^r}{\overset{k_{16}^f}{\rightleftarrows}} AX \xrightarrow{K_{17}} A^* + X^*$	*iPFB Rx 11*
$B + Y^* \underset{k_{18}^r}{\overset{k_{18}^f}{\rightleftarrows}} BY \xrightarrow{K_{19}} B^* + Y^*$	*iPFB Rx 12*

Supplementary Table I: Reaction networks describing the dynamics of the signaling motif. The ligand L activates the input source from the inactive (I) to the active (I^*) state. The active input source I* can then activate intermediate molecules (A^*, B^*), which can further activate downstream components (X^*, Y^*). IL, AI, BI, XA, YB, IX, IY, XI, YI, YA, XB, AX, and BY are

complex signaling molecules that are formed from association of the various motif components.

All active molecules are assumed to be spontaneously deactivated.

SM2. Supplementary Table II

Linear motif core structure ODE model

$d[L]/dt = -k_1^f[I][L] + (k_1^r + K_2)[IL]$	*LM Eq1*
$d[I]/dt = -k_1^f[I][L] + k_1^r[IL] + K_3[I^*]$	*LM Eq2*
$d[I^*]/dt = K_2[IL] - K_3[I^*] - k_4^f[A][I^*] + (k_4^r + K_5)[AI] - k_7^f[B][I^*] + (k_7^r + K_8)[BI]$	*LM Eq3*
$d[A]/dt = -k_4^f[A][I^*] + k_4^r[AI] + K_6[A^*]$	*LM Eq4*
$d[A^*]/dt = K_5[AI] - K_6[A^*] - k_{10}^f[X][A^*] + (k_{10}^r + K_{11})[XA]$	*LM Eq5*
$d[B]/dt = -k_7^f[B][I^*] + k_7^r[BI] + K_9[B^*]$	*LM Eq6*
$d[B^*]/dt = K_8[BI] - K_9[B^*] - k_{13}^f[Y][B^*] + (k_{13}^r + K_{14})[YB]$	*LM Eq7*
$d[X]/dt = -k_{10}^f[X][A^*] + k_{10}^r[XA] + K_{12}[X^*]$	*LM Eq8*
$d[X^*]/dt = K_{11}[XA] - K_{12}[X^*]$	*LM Eq9*
$d[Y]/dt = -k_{13}^f[Y][B^*] + k_{13}^r[YB] + K_{15}[Y^*]$	*LM Eq10*
$d[Y^*]/dt = K_{14}[YB] - K_{15}[Y^*]$	*LM Eq11*
$d[IL]/dt = k_1^f[I][L] - (k_1^r + K_2)[IL]$	*LM Eq12*
$d[AI]/dt = k_4^f[A][I^*] - (k_4^r + K_5)[AI]$	*LM Eq13*
$d[BI]/dt = k_7^f[B][I^*] - (k_7^r + K_8)[BI]$	*LM Eq14*
$d[XA]/dt = k_{10}^f[X][A^*] - (k_{10}^r + K_{11})[XA]$	*LM Eq15*
$d[YB]/dt = k_{13}^f[Y][B^*] - (k_{13}^r + K_{14})[YB]$	*LM Eq16*

Negative feedback modified and additional ODEs

$d[I]/dt = -k_1^f[I][L] + k_1^r[IL] + K_3[I^*] + K_{17}[IX] + K_{19}[IY]$	*NFB Eq2*
$d[I^*]/dt = K_2[IL] - K_3[I^*] - k_4^f[A][I^*] + (k_4^r + K_5)[AI] - k_7^f[B][I^*] + (k_7^r + K_8)[BI] - k_{16}^f[I^*][X^*] + k_{16}^r[IX] - k_{18}^f[I^*][Y^*] + k_{18}^r[IY]$	*NFB Eq3*
$d[X^*]/dt = K_{11}[XA] - K_{12}[X^*] - k_{16}^f[I^*][X^*] + (k_{16}^r + K_{17})[IX]$	*NFB Eq9*
$d[Y^*]/dt = K_{14}[YB] - K_{15}[Y^*] - k_{18}^f[I^*][Y^*] + (k_{18}^r + K_{19})[IY]$	*NFB Eq11*
$d[IX]/dt = k_{16}^f[I^*][X^*] - (k_{16}^r + K_{17})[IX]$	*NFB Eq17*
$d[IY]/dt = k_{18}^f[I^*][Y^*] - (k_{18}^r + K_{19})[IY]$	*NFB Eq18*

Positive feedback modified and additional ODEs

$d[I]/dt = -k_1^f[I][L] + k_1^r[IL] + K_3[I^*] - k_{16}^f[I][X^*] + k_{16}^r[IX] - k_{18}^f[I][Y^*] + k_{18}^r[IY]$	*PFB Eq2*

$$d\left[I^*\right]/dt = K_2\left[IL\right] - K_3\left[I^*\right] - k_4^f\left[A\right]\left[I^*\right] + \left(k_4^r + K_5\right)\left[AI\right] - k_7^f\left[B\right]\left[I^*\right] + \\ + \left(k_7^r + K_8\right)\left[BI\right] + K_{17}\left[IX\right] + K_{19}\left[IY\right] \qquad \textit{PFB Eq3}$$

$$d\left[X^*\right]/dt = K_{11}\left[XA\right] - K_{12}\left[X^*\right] - k_{16}^f\left[I\right]\left[X^*\right] + \left(k_{16}^r + K_{17}\right)\left[IX\right] \qquad \textit{PFB Eq9}$$

$$d\left[Y^*\right]/dt = K_{14}\left[YB\right] - K_{15}\left[Y^*\right] - k_{18}^f\left[I\right]\left[Y^*\right] + \left(k_{18}^r + K_{19}\right)\left[IY\right] \qquad \textit{PFB Eq11}$$

$$d\left[IX\right]/dt = k_{16}^f\left[I\right]\left[X^*\right] - \left(k_{16}^r + K_{17}\right)\left[IX\right] \qquad \textit{PFB Eq17}$$

$$d\left[IY\right]/dt = k_{18}^f\left[I\right]\left[Y^*\right] - \left(k_{18}^r + K_{19}\right)\left[IY\right] \qquad \textit{PFB Eq18}$$

Positive-negative feedback modified and additional ODEs

$$d\left[I\right]/dt = -k_1^f\left[I\right]\left[L\right] + k_1^r\left[IL\right] + K_3\left[I^*\right] - k_{16}^f\left[I\right]\left[X^*\right] + k_{16}^r\left[IX\right] + K_{19}\left[IY\right] \qquad \textit{PNFB Eq2}$$

$$d\left[I^*\right]/dt = K_2\left[IL\right] - K_3\left[I^*\right] - k_4^f\left[A\right]\left[I^*\right] + \left(k_4^r + K_5\right)\left[AI\right] - k_7^f\left[B\right]\left[I^*\right] + \\ + \left(k_7^r + K_8\right)\left[BI\right] + K_{17}\left[IX\right] - k_{18}^f\left[I^*\right]\left[Y^*\right] + k_{18}^r\left[IY\right] \qquad \textit{PNFB Eq3}$$

$$d\left[X^*\right]/dt = K_{11}\left[XA\right] - K_{12}\left[X^*\right] - k_{16}^f\left[I\right]\left[X^*\right] + \left(k_{16}^r + K_{17}\right)\left[IX\right] \qquad \textit{PNFB Eq9}$$

$$d\left[Y^*\right]/dt = K_{14}\left[YB\right] - K_{15}\left[Y^*\right] - k_{18}^f\left[I^*\right]\left[Y^*\right] + \left(k_{18}^r + K_{19}\right)\left[IY\right] \qquad \textit{PNFB Eq11}$$

$$d\left[IX\right]/dt = k_{16}^f\left[I\right]\left[X^*\right] - \left(k_{16}^r + K_{17}\right)\left[IX\right] \qquad \textit{PNFB Eq17}$$

$$d\left[IY\right]/dt = k_{18}^f\left[I^*\right]\left[Y^*\right] - \left(k_{18}^r + K_{19}\right)\left[IY\right] \qquad \textit{PNFB Eq18}$$

Negative feedforward modified and additional ODEs

$$d\left[I^*\right]/dt = K_2\left[IL\right] - K_3\left[I^*\right] - k_4^f\left[A\right]\left[I^*\right] + \left(k_4^r + K_5\right)\left[AI\right] - k_7^f\left[B\right]\left[I^*\right] + \\ + \left(k_7^r + K_8\right)\left[BI\right] - k_{16}^f\left[X^*\right]\left[I^*\right] + \left(k_{16}^r + K_{17}\right)\left[IX\right] - k_{18}^f\left[Y^*\right]\left[I^*\right] + \left(k_{18}^r + K_{19}\right)\left[IY\right] \qquad \textit{NFF Eq3}$$

$$d\left[X\right]/dt = -k_{10}^f\left[X\right]\left[A^*\right] + k_{10}^r\left[XA\right] + K_{12}\left[X^*\right] + K_{17}\left[IX\right] \qquad \textit{NFF Eq8}$$

$$d\left[X^*\right]/dt = K_{11}\left[XA\right] - K_{12}\left[X^*\right] - k_{16}^f\left[X^*\right]\left[I^*\right] + k_{16}^r\left[IX\right] \qquad \textit{NFF Eq9}$$

$$d\left[Y\right]/dt = -k_{13}^f\left[Y\right]\left[B^*\right] + k_{13}^r\left[YB\right] + K_{15}\left[Y^*\right] + K_{19}\left[IY\right] \qquad \textit{NFF Eq10}$$

$$d\left[Y^*\right]/dt = K_{14}\left[YB\right] - K_{15}\left[Y^*\right] - k_{18}^f\left[Y^*\right]\left[I^*\right] + k_{18}^r\left[IY\right] \qquad \textit{NFF Eq11}$$

$$d\left[XI\right]/dt = k_{16}^f\left[X^*\right]\left[I^*\right] - \left(k_{16}^r + K_{17}\right)\left[XI\right] \qquad \textit{NFF Eq17}$$

Positive feedforward modified and additional ODEs

$$d\left[I^*\right]/dt = K_2\left[IL\right] - K_3\left[I^*\right] - k_4^f\left[A\right]\left[I^*\right] + \left(k_4^r + K_5\right)\left[AI\right] - k_7^f\left[B\right]\left[I^*\right] + \\ + \left(k_7^r + K_8\right)\left[BI\right] - k_{16}^f\left[X\right]\left[I^*\right] + \left(k_{16}^r + K_{17}\right)\left[IX\right] - k_{18}^f\left[Y\right]\left[I^*\right] + \left(k_{18}^r + K_{19}\right)\left[IY\right] \qquad \textit{PFF Eq3}$$

$$d\left[X\right]/dt = -k_{10}^f\left[X\right]\left[A^*\right] + k_{10}^r\left[XA\right] + K_{12}\left[X^*\right] - k_{16}^f\left[X\right]\left[I^*\right] + k_{16}^r\left[IX\right] \qquad \textit{PFF Eq8}$$

$$d\left[X^*\right]/dt = K_{11}\left[XA\right] - K_{12}\left[X^*\right] + K_{17}\left[IX\right] \qquad \textit{PFF Eq9}$$

$$d\left[Y\right]/dt = -k_{13}^f\left[Y\right]\left[B^*\right] + k_{13}^r\left[YB\right] + K_{15}\left[Y^*\right] - k_{18}^f\left[Y\right]\left[I^*\right] + k_{18}^r\left[IY\right] \qquad \textit{PFF Eq10}$$

$$d\left[Y^*\right]/dt = K_{14}\left[YB\right] - K_{15}\left[Y^*\right] + K_{19}\left[IY\right] \qquad \textit{PFF Eq11}$$

$d\left[XI\right]/dt = k_{16}^{f}\left[X\right]\left[I^{*}\right] - \left(k_{16}^{r} + K_{17}\right)\left[XI\right]$ *PFF Eq17*

Positive-negative feedforward modified and additional ODEs

$d\left[I^{*}\right]/dt = K_{2}\left[IL\right] - K_{3}\left[I^{*}\right] - k_{4}^{f}\left[A\right]\left[I^{*}\right] + \left(k_{4}^{r} + K_{5}\right)\left[AI\right] - k_{7}^{f}\left[B\right]\left[I^{*}\right] + + \left(k_{7}^{r} + K_{8}\right)\left[BI\right] - k_{16}^{f}\left[X\right]\left[I^{*}\right] + \left(k_{16}^{r} + K_{17}\right)\left[IX\right] - k_{18}^{f}\left[Y^{*}\right]\left[I^{*}\right] + \left(k_{18}^{r} + K_{19}\right)\left[IY\right]$ *PNFF Eq3*

$d\left[X\right]/dt = -k_{10}^{f}\left[X\right]\left[A^{*}\right] + k_{10}^{r}\left[XA\right] + K_{12}\left[X^{*}\right] - k_{16}^{f}\left[X\right]\left[I^{*}\right] + k_{16}^{r}\left[IX\right]$ *PNFF Eq8*

$d\left[X^{*}\right]/dt = K_{11}\left[XA\right] - K_{12}\left[X^{*}\right] + K_{17}\left[IX\right]$ *PNFF Eq9*

$d\left[Y\right]/dt = -k_{13}^{f}\left[Y\right]\left[B^{*}\right] + k_{13}^{r}\left[YB\right] + K_{15}\left[Y^{*}\right] + K_{19}\left[IY\right]$ *PNFF Eq10*

$d\left[Y^{*}\right]/dt = K_{14}\left[YB\right] - K_{15}\left[Y^{*}\right] - k_{18}^{f}\left[Y^{*}\right]\left[I^{*}\right] + k_{18}^{r}\left[IY\right]$ *PNFF Eq11*

$d\left[XI\right]/dt = k_{16}^{f}\left[X\right]\left[I^{*}\right] - \left(k_{16}^{r} + K_{17}\right)\left[XI\right]$ *PNFF Eq17*

Coherent bifan modified and additional ODEs

$d\left[A^{*}\right]/dt = K_{5}\left[AI\right] - K_{6}\left[A^{*}\right] - k_{10}^{f}\left[X\right]\left[A^{*}\right] + \left(k_{10}^{r} + K_{11}\right)\left[XA\right] - k_{16}^{f}\left[Y\right]\left[A^{*}\right] + \left(k_{16}^{r} + K_{17}\right)\left[YA\right]$ *CB Eq5*

$d\left[B^{*}\right]/dt = K_{8}\left[BI\right] - K_{9}\left[B^{*}\right] - k_{13}^{f}\left[Y\right]\left[B^{*}\right] + \left(k_{13}^{r} + K_{14}\right)\left[YB\right] - k_{18}^{f}\left[X\right]\left[B^{*}\right] + \left(k_{18}^{r} + K_{19}\right)\left[XB\right]$ *CB Eq7*

$d\left[X\right]/dt = -k_{10}^{f}\left[X\right]\left[A^{*}\right] + k_{10}^{r}\left[XA\right] + K_{12}\left[X^{*}\right] - k_{18}^{f}\left[X\right]\left[B^{*}\right] + k_{18}^{r}\left[XB\right]$ *CB Eq8*

$d\left[X^{*}\right]/dt = K_{11}\left[XA\right] - K_{12}\left[X^{*}\right] + K_{19}\left[XB\right]$ *CB Eq9*

$d\left[Y\right]/dt = -k_{13}^{f}\left[Y\right]\left[B^{*}\right] + k_{13}^{r}\left[YB\right] + K_{15}\left[Y^{*}\right] - k_{16}^{f}\left[Y\right]\left[A^{*}\right] k_{16}^{r}\left[YA\right]$ *CB Eq10*

$d\left[Y^{*}\right]/dt = K_{14}\left[YB\right] - K_{15}\left[Y^{*}\right] + K_{17}\left[YA\right]$ *CB Eq11*

$d\left[YA\right]/dt = k_{16}^{f}\left[Y\right]\left[A^{*}\right] - \left(k_{16}^{r} + K_{17}\right)\left[YA\right]$ *CB Eq17*

Incoherent bifan modified and additional ODEs

$d\left[A^{*}\right]/dt = K_{5}\left[AI\right] - K_{6}\left[A^{*}\right] - k_{10}^{f}\left[X\right]\left[A^{*}\right] + \left(k_{10}^{r} + K_{11}\right)\left[XA\right] - k_{16}^{f}\left[Y^{*}\right]\left[A^{*}\right] + \left(k_{16}^{r} + K_{17}\right)\left[YA\right]$ *IB Eq5*

$d\left[B^{*}\right]/dt = K_{8}\left[BI\right] - K_{9}\left[B^{*}\right] - k_{13}^{f}\left[Y\right]\left[B^{*}\right] + \left(k_{13}^{r} + K_{14}\right)\left[YB\right] - k_{18}^{f}\left[X^{*}\right]\left[B^{*}\right] + \left(k_{18}^{r} + K_{19}\right)\left[XB\right]$ *IB Eq7*

$d\left[X\right]/dt = -k_{10}^{f}\left[X\right]\left[A^{*}\right] + k_{10}^{r}\left[XA\right] + K_{12}\left[X^{*}\right] + K_{19}\left[XB\right]$ *IB Eq8*

$d\left[X^{*}\right]/dt = K_{11}\left[XA\right] - K_{12}\left[X^{*}\right] - k_{18}^{f}\left[X^{*}\right]\left[B^{*}\right] + k_{18}^{r}\left[XB\right]$ *IB Eq9*

$d\left[Y\right]/dt = -k_{13}^{f}\left[Y\right]\left[B^{*}\right] + k_{13}^{r}\left[YB\right] + K_{15}\left[Y^{*}\right] + K_{17}\left[YA\right]$ *IB Eq10*

$d\left[Y^{*}\right]/dt = K_{14}\left[YB\right] - K_{15}\left[Y^{*}\right] - k_{16}^{f}\left[Y^{*}\right]\left[A^{*}\right] k_{16}^{r}\left[YA\right]$ *IB Eq11*

$d\left[YA\right]/dt = k_{16}^{f}\left[Y^{*}\right]\left[A^{*}\right] - \left(k_{16}^{r} + K_{17}\right)\left[YA\right]$ *IB Eq17*

Partially coherent bifan modified and additional ODEs

$d\left[A^{*}\right]/dt = K_{5}\left[AI\right] - K_{6}\left[A^{*}\right] - k_{10}^{f}\left[X\right]\left[A^{*}\right] + \left(k_{10}^{r} + K_{11}\right)\left[XA\right] - k_{16}^{f}\left[Y\right]\left[A^{*}\right] + \left(k_{16}^{r} + K_{17}\right)\left[YA\right]$ *PCB Eq5*

$d\left[B^{*}\right]/dt = K_{8}\left[BI\right] - K_{9}\left[B^{*}\right] - k_{13}^{f}\left[Y\right]\left[B^{*}\right] + \left(k_{13}^{r} + K_{14}\right)\left[YB\right] - k_{18}^{f}\left[X^{*}\right]\left[B^{*}\right] + \left(k_{18}^{r} + K_{19}\right)\left[XB\right]$ *PCB Eq7*

$d\left[X\right]/dt = -k_{10}^{f}\left[X\right]\left[A^{*}\right] + k_{10}^{r}\left[XA\right] + K_{12}\left[X^{*}\right] + K_{19}\left[XB\right]$ *PCB Eq8*

$d\left[X^*\right]/dt = K_{11}[XA] - K_{12}\left[X^*\right] - k_{18}^f\left[X^*\right]\left[B^*\right] + k_{18}^r[XB]$	*PCB Eq9*
$d[Y]/dt = -k_{13}^f[Y]\left[B^*\right] + k_{13}^r[YB] + K_{15}\left[Y^*\right] - k_{16}^f[Y]\left[A^*\right]k_{16}^r[YA]$	*PCB Eq10*
$d\left[Y^*\right]/dt = K_{14}[YB] - K_{15}\left[Y^*\right] + K_{17}[YA]$	*PCB Eq11*
$d[YA]/dt = k_{16}^f[Y]\left[A^*\right] - \left(k_{16}^r + K_{17}\right)[YA]$	*PCB Eq17*
Isolated negative feedback modified and additional ODEs	
$d[A]/dt = -k_4^f[A]\left[I^*\right] + k_4^r[AI] + K_6\left[A^*\right] + K_{17}[AX]$	*iNFB Eq4*
$d\left[A^*\right]/dt = K_5[AI] - K_6\left[A^*\right] - k_{10}^f[X]\left[A^*\right]\left(k_{10}^r + K_{11}\right)[XA] - k_{16}^f\left[A^*\right]\left[X^*\right] + k_{16}^r[AX]$	*iNFB Eq5*
$d[B]/dt = -k_7^f[B]\left[I^*\right] + k_7^r[BI] + K_9\left[B^*\right] + K_{19}[BY]$	*iNFB Eq6*
$d\left[B^*\right]/dt = K_8[BI] - K_9\left[B^*\right] - k_{13}^f[Y]\left[B^*\right] + \left(k_{13}^r + K_{14}\right)[YB] - k_{18}^f\left[B^*\right]\left[Y^*\right] + k_{18}^r[BY]$	*iNFB Eq7*
$d\left[X^*\right]/dt = -k_{16}^f\left[A^*\right]\left[X^*\right] + \left(k_{16}^r + K_{17}\right)[AX]$	*iNFB Eq9*
$d\left[Y^*\right]/dt = -k_{18}^f\left[B^*\right]\left[Y^*\right] + \left(k_{18}^r + K_{19}\right)[BY]$	*iNFB Eq11*
$d[AX]/dt = k_{16}^f\left[A^*\right]\left[X^*\right] - \left(k_{16}^r + K_{17}\right)[AX]$	*iNFB Eq17*
$d[BY]/dt = k_{18}^f\left[B^*\right]\left[Y^*\right] - \left(k_{18}^r + K_{19}\right)[BY]$	*iNFB Eq18*
Isolated positive feedback modified and additional ODEs	
$d[A]/dt = -k_4^f[A]\left[I^*\right] + k_4^r[AI] + K_6\left[A^*\right] - k_{16}^f[A]\left[X^*\right] + k_{16}^r[AX]$	*iPFB Eq 4*
$d\left[A^*\right]/dt = K_5[AI] - K_6\left[A^*\right] - k_{10}^f[X]\left[A^*\right] + \left(k_{10}^r + K_{11}\right)[XA] + K_{17}[AX]$	*iPFB Eq 5*
$d[B]/dt = -k_7^f[B]\left[I^*\right] + k_7^r[BI] + K_9\left[B^*\right] - k_{18}^f[B]\left[Y^*\right] + k_{18}^r[BY]$	*iPFB Eq 6*
$d\left[B^*\right]/dt = K_8[BI] - K_9\left[B^*\right] - k_{13}^f[Y]\left[B^*\right] + \left(k_{13}^r + K_{14}\right)[YB] + K_{19}[BY]$	*iPFB Eq 7*
$d\left[X^*\right]/dt = -k_{16}^f[A]\left[X^*\right] + \left(k_{16}^r + K_{17}\right)[AX]$	*iPFB Eq 9*
$d\left[Y^*\right]/dt = -k_{18}^f[B]\left[Y^*\right] + \left(k_{18}^r + K_{19}\right)[BY]$	*iPFB Eq 11*
$d[AX]/dt = k_{16}^f[A]\left[X^*\right] - \left(k_{16}^r + K_{17}\right)[AX]$	*iPFB Eq17*
$d[BY]/dt = k_{18}^f[B]\left[Y^*\right] - \left(k_{18}^r + K_{19}\right)[BY]$	*iPFB Eq18*

Supplementary Table II: Ordinary differential equation (ODE) modeling describing the dynamics of the signaling motif. The components in square brackets represent the concentrations of the signaling species, and the k's represent the kinetic rate constants.

SM3. Supplementary Table III

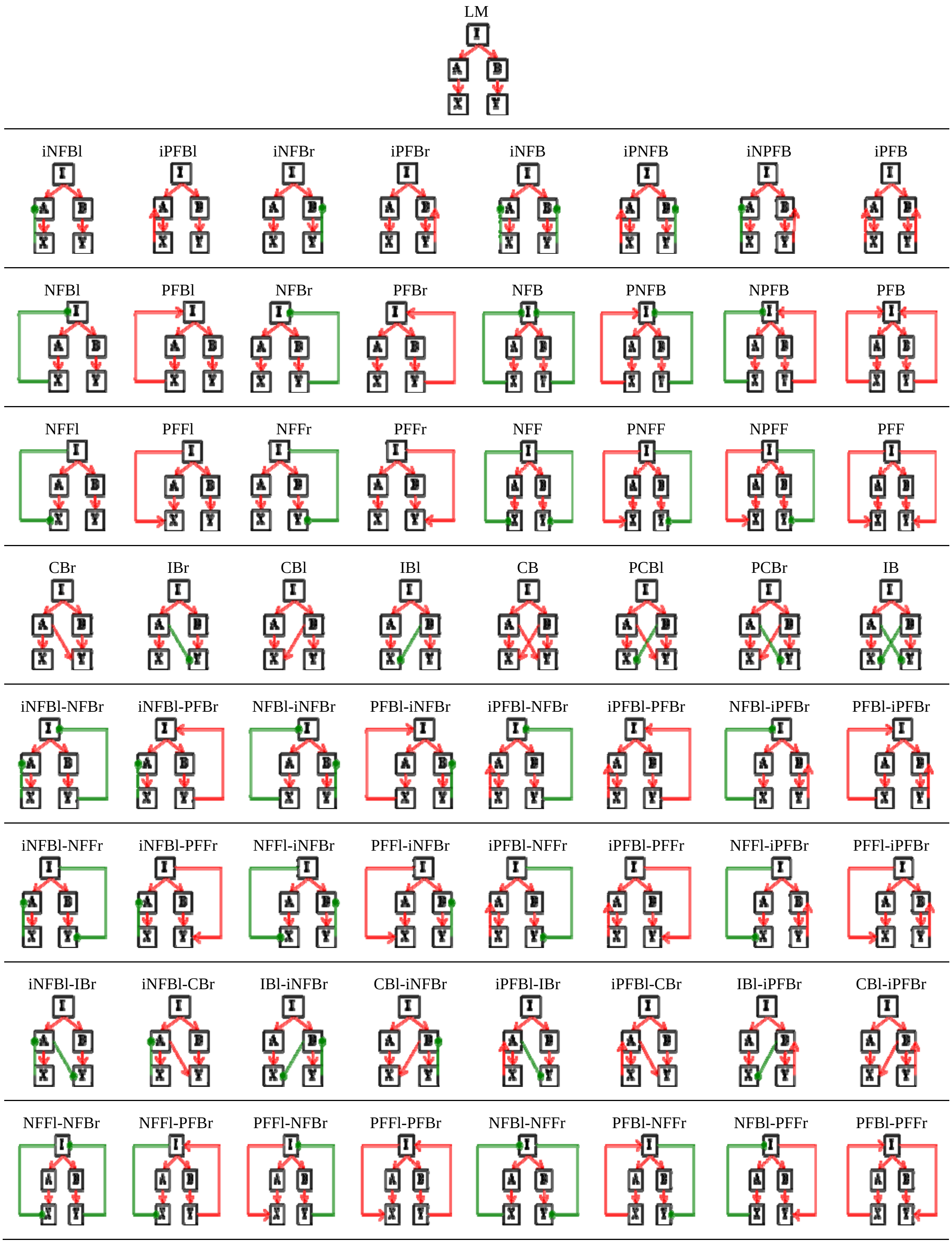

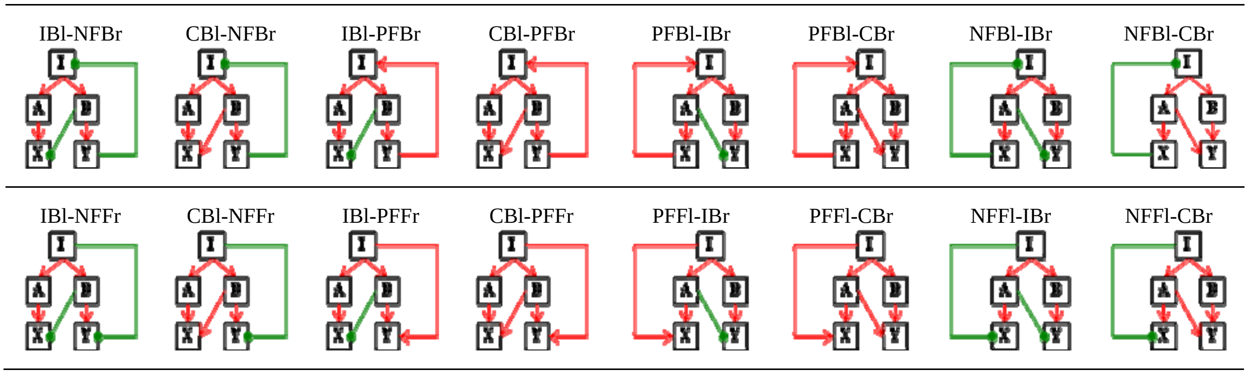

Supplementary Table III: Motif topologies that interconnect the linear, feedback, feed-forward, bifan, and isolated feedback motifs.

SM4. Supplementary Figure 1

A

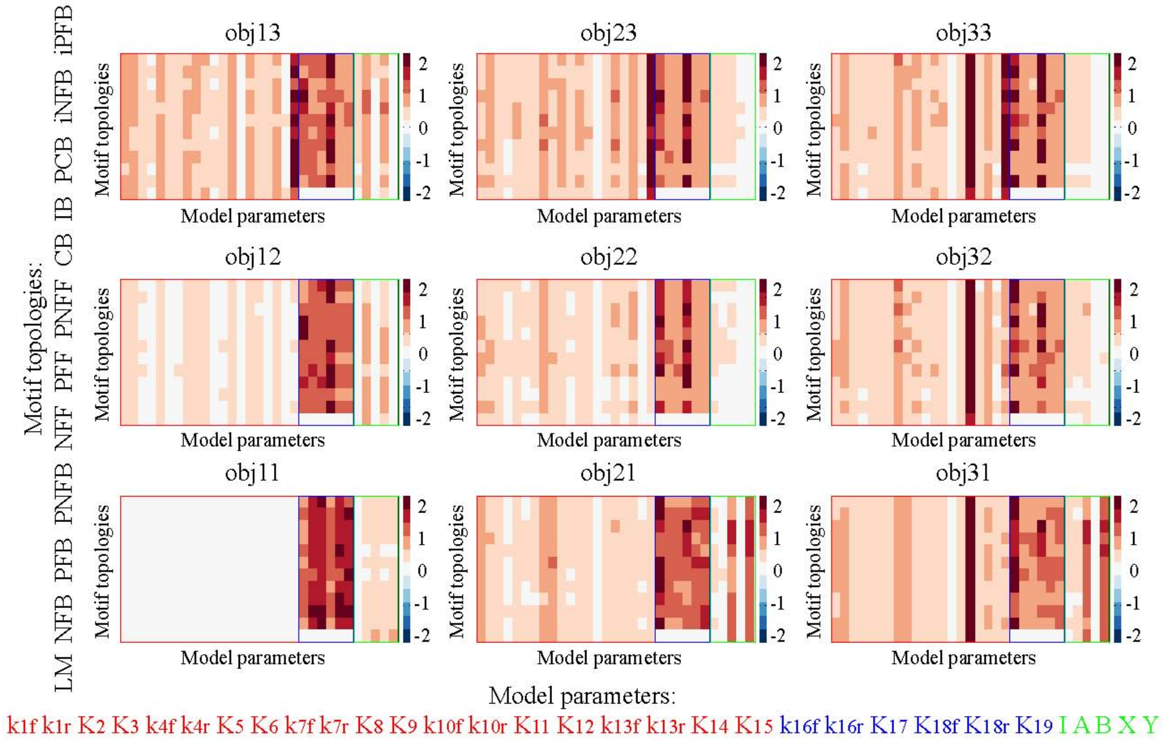

B

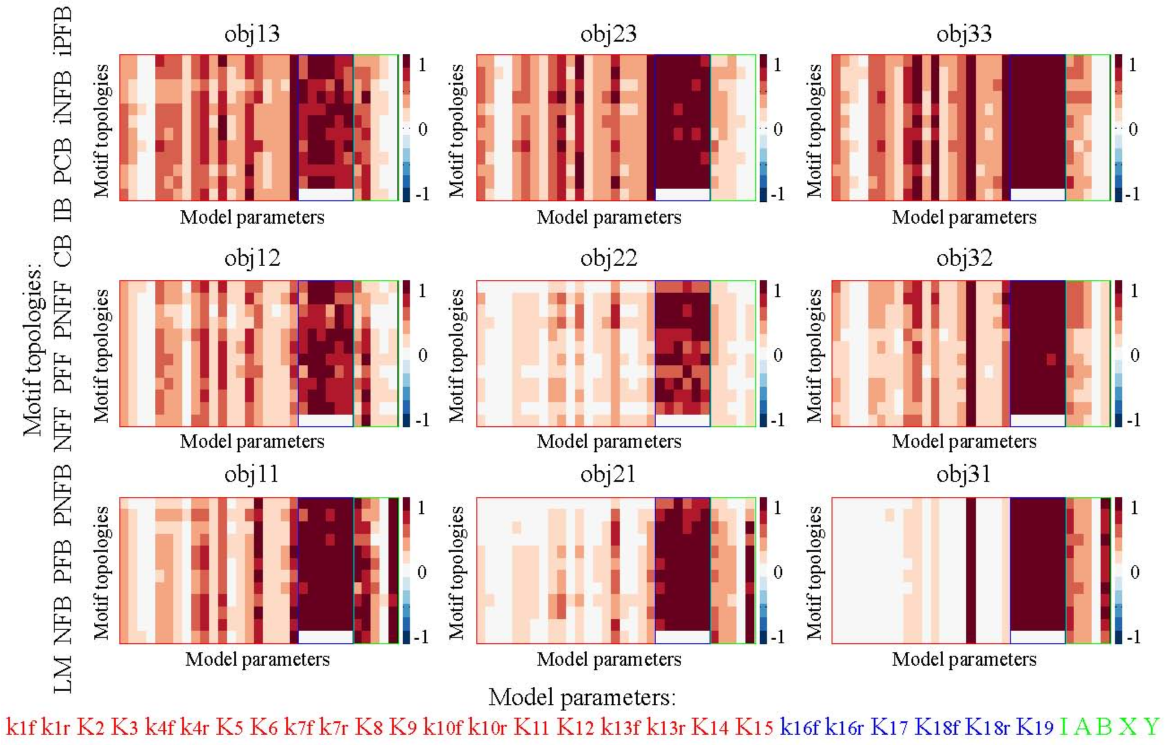

C

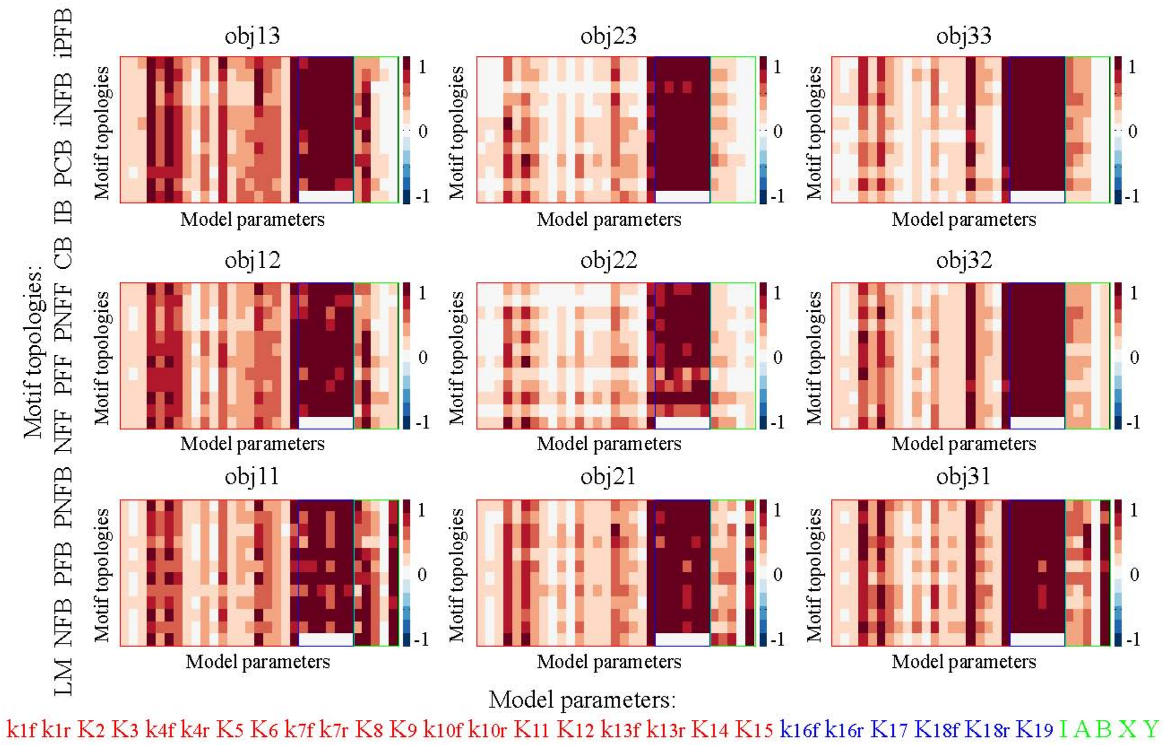

Supplementary Figure 1: Motif tunability to static output objectives obtained through PSO sampling of model parameters necessary for ODE implementation. Particle positions were initialized to points A) $xy_{1.5\_8.5}$; B) $xy_{8.5\_1.5}$; and C) $xy_{8.5\_8.5}$.

SM5. Supplementary Figure 2

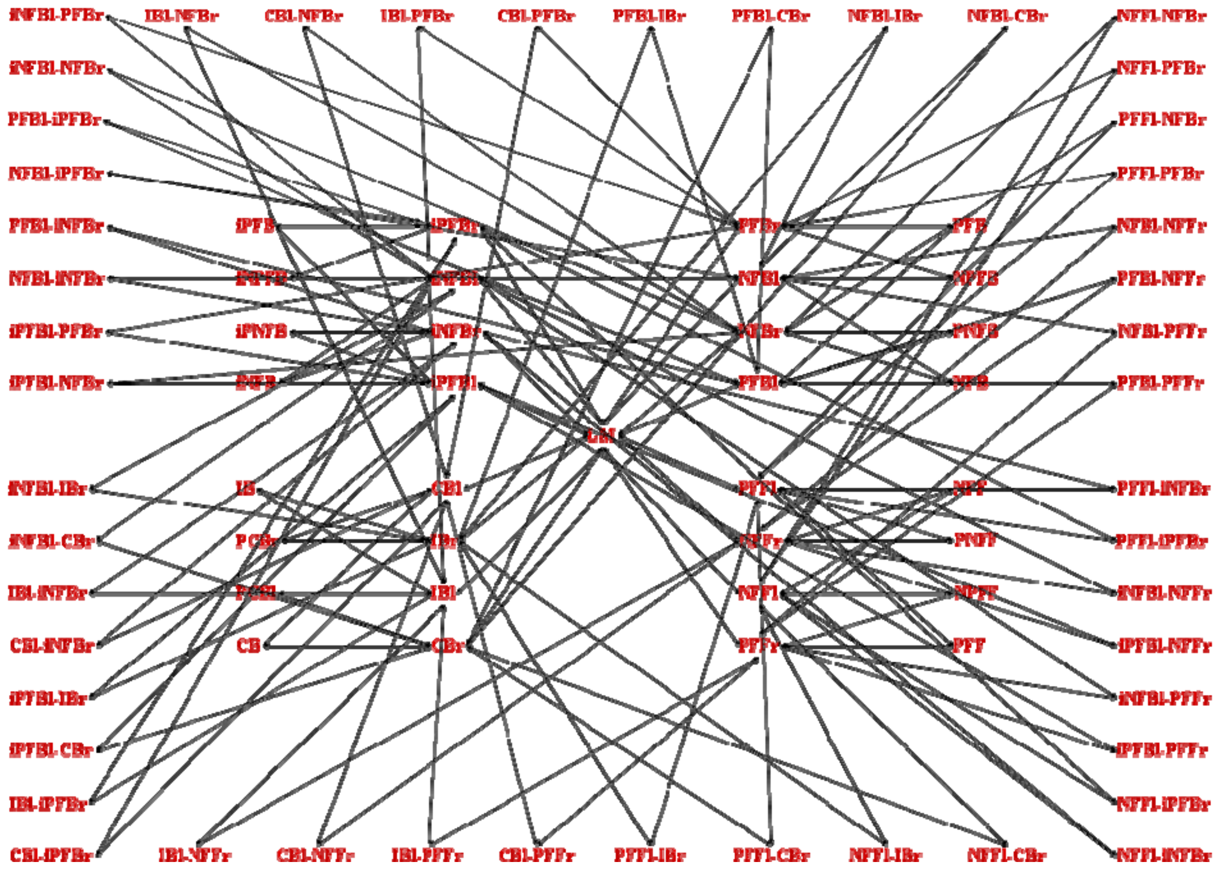

Supplementary Figure 2: Interaction network of 81 motif topologies that linked the linear, feedback, feed-forward, bifan, and isolated feedback motifs.
